# A CARE-compliant article: A case report of unusual eschar and extensive soft tissue necrosis in Tsutsugamushi disease

**DOI:** 10.1097/MD.0000000000036009

**Published:** 2023-11-10

**Authors:** Je Yeon Byeon, Hyun Kim, Da Woon Lee, Hwan Jun Choi

**Affiliations:** a Department of Plastic and Reconstructive Surgery, College of Medicine, Soonchunhyang University Cheonan Hospital, Cheonan, South Korea; b Institute of Tissue Regeneration, College of Medicine, Soonchunhyang University, Cheonan, South Korea.

**Keywords:** case report, eschar, infection, necrosis, scrub typhus

## Abstract

**Rationale:**

Tsutsugamushi disease is a common infectious disease in the Northern Hemisphere. A patient infected with tsutsugamushi disease will show a characteristic clinical course with eschar formation, which is mostly small and self-limited in nature without causing major problems. We report a rare case of unusually extensive necrosis started from a small eschar.

**Patient concerns:**

In this report, a 65-year-old female patient with a history of diabetes mellitus present an 8 × 6 cm-sized huge eschar and extensive soft tissue necrosis aggravated from a small eschar. Also, there were 3 other small eschars in the scalp and left flank area. In early July, she was farming in a field in Hongseong-gun, South Korea. She had been treated at another hospital for 2 weeks. However, the eschar became bigger and worse.

**Diagnoses:**

After admission, escharectomy was performed and extensive soft tissue necrosis was identified. Orientia tsutsugamushi antibody tests were positive from blood test. Providencia rettgeri and Enterococcus faecalis were detected in a tissue bacterial culture test.

**Intervention:**

While using oral azithromycin and intravenous imipenem/cilastatin, the necrosis of the thigh was excised and covered by lateral femoral circumflex artery based myocutaneous Keystone flap.

**Outcomes:**

The remaining small eschars recovered spontaneously, the large eschars that had caused necrosis were successfully treated, and all other clinical symptoms improved without complications.

**Lessons:**

For unusual eschar of an unknown cause, especially in patients with uncontrolled diabetes or immunocompromised, the possibility of Tsutsugamushi should be considered. Careful physical examination and proper management should be performed as soon as possible.

## 1. Introduction

Changes in the pattern of infectious diseases caused by climate change are major global issues.^[1]^ There are many species of parasitic mites in a variety of habitats worldwide. Eutrombicula alfreddugesi in the south of the United States, Trombicula autumnalis in Europe, and species of the Leptotrombidium genus in Asia and Oceania are species most commonly referred to as chiggers.^[2]^ Tsutsugamushi disease, or scrub typhus is caused by *Orientia tsutsugamushi*. It is mainly transmitted by chigger mites.^[3]^ It is known to occur not only in the Asian-pacific area and northern Australia but also in Africa, southern Chile, and the middle east.^[4]^ It occurs mainly in summer and autumn from June to September in the Northern Hemisphere. It is known to infect rural areas and semi-urban environments.^[5,6]^ A clinical course with headache, anorexia, and malaise characterizes this disease. It is usually slow in onset.^[7]^ Abrupt onset of high fever, intense headache, and diffuse myalgias can also occur.^[8]^ In addition, a rash that appears at the site of chigger bites in South Korea most often leads to eschar formation with a size of 5 to 20 mm.^[8–10]^ Its clinical courses are usually self-limited, and the eschar will disappear when the scab falls off.^[5]^ It is rare for the eschar of Tsutsugamushi to cause extensive tissue necrosis. We report a rare case of extensive necrosis involving the muscle layer that started from a small eschar of Tsutsugamushi disease.

## 2. Patients and Methods

This retrospective case study involved a patient who visited our outpatient clinic, Department of Plastic and Reconstructive Surgery. The study protocol was approved by the Institutional Review Board (number:2023-06-044). The written informed consent was obtained from the patient for publication of this case report details. All procedures were performed in accordance with the ethical standards of the institutional and/or national research committee, and the 1964 Declaration of Helsinki and its later amendments or comparable ethical standards.

## 3. Case report

**Figure 1.**
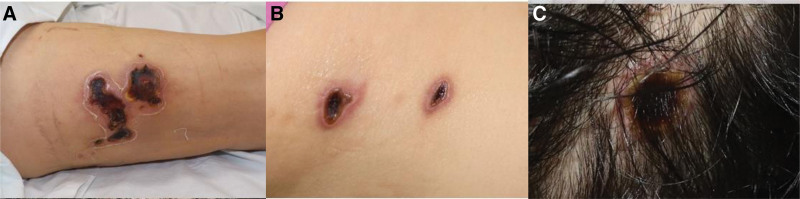
Initial photographic finding. (A) A large eschar is formed in the patient’s right thigh with induration and heating sensation. (B) Two eschars in the left flank area. (C) An eschar in the scalp area.

A 65-year-old Asian woman with a history of diabetes mellitus and hypertension presented to the hospital with a huge eschar and extensive right thigh infection (Fig. 1). She complained of no other symptoms except pain and a heating sensation around the eschar on the right thigh. She had been treated at another hospital for 2 weeks for a small eschar on her thigh. However, there was no improvement. During history taking, it was revealed that she had been working in the agricultural field 3 weeks prior. In early July, she was farming in a field in Hongseong-gun, South Korea. The patient denied any family history of similar symptoms. Three other small eschars were found on her left flank and scalp (Fig. 1). The size of the eschar located on the scalp was 7 × 7 mm. Two eschars on the left flank were 11 × 7 mm and 10 × 4 cm in size. These eschars were accompanied by some redness around them. However, there was no sign of a severe infection. A 8 × 6 cm-sized eschar was observed at the anterior side of the right thigh. Infectious signs such as tenderness, induration, and heating sensation were observed around the eschar of the right thigh. Otherwise, there were no specific abnormalities in other areas.

**Figure 2.**
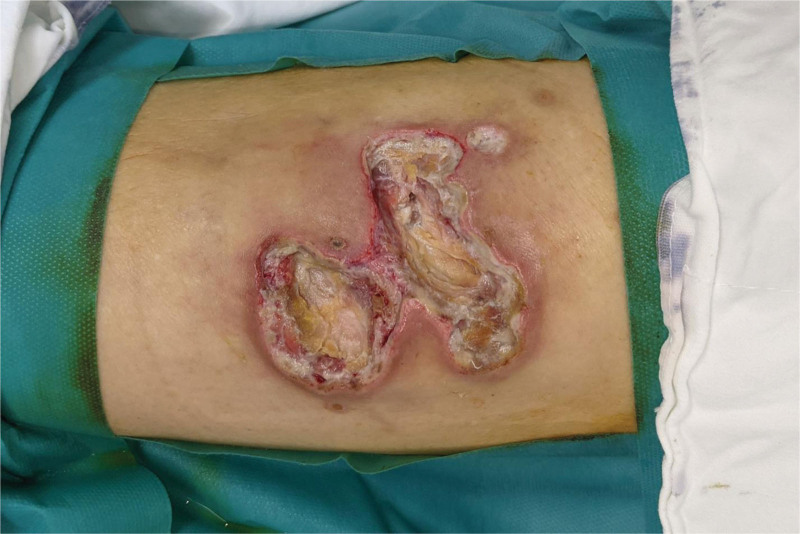
Preoperative photographic finding. After escharectomy, necrotic tissues are extended to the muscle layer beyond the soft tissue.

**Figure 3.**
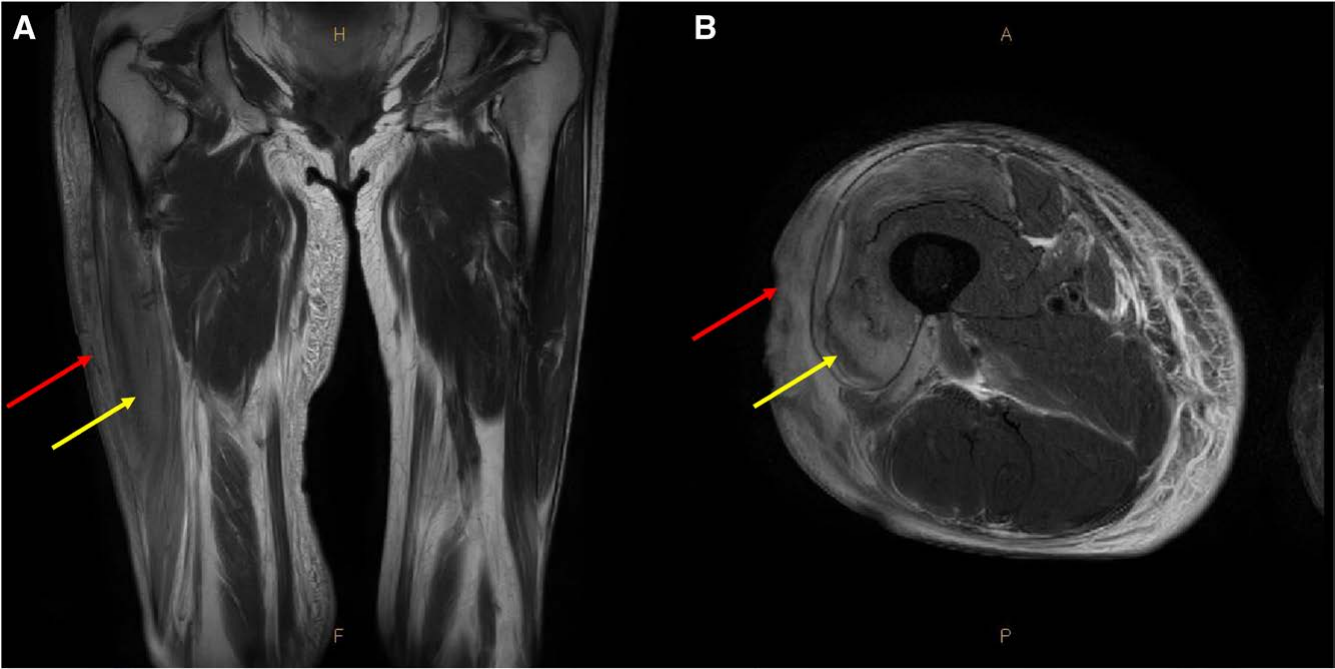
Initial magnetic resonance imaging showing necrosis of soft tissue. T2 image of the MRI performed at the time of admission revealed some necrosis of the vastus lateralis muscle, including the subcutaneous layer below the ulcerative lesion of the skin. Low attenuation was seen in necrotic muscle and fat. The red arrow points to the location of the eschar. The yellow arrow indicates a necrotic muscle. (A) Coronal view of T2 image. (B) Axial view of T2 image. MRI = magnetic resonance imaging.

At the time of admission, the blood test showed an elevated erythrocyte sedimentation rate of 77 mm/hr, a C-reactive protein of 113 mg/L, and an elevated glycated hemoglobin of 11.7%. There were no other abnormalities in blood counts. The *O tsutsugamushi* antibody test was performed, and a positive result was confirmed. Lyme disease, Rickettsia, tuberculosis, Hantaan virus, and leptospira were all negative. During the hospital stay, her systolic blood pressure was 110 to 130 mm Hg, and her diastolic blood pressure was 70 to 80 mm Hg, which was in their normal ranges. Her resting heart rate was also in the normal range of 66 to 82 beats. Other organs, such as the liver, lungs, and kidneys, also function normally. However, as with glycated hemoglobin, which was high at the time of hospitalization, blood sugar level was not well controlled during the hospital stay. Due to the patient’s uncooperative attitude, dietary control and insulin administration could have been better achieved. After the escharectomy, it was confirmed that necrosis had progressed under the muscle level. The necrotic tissue was excised for histological examination and bacterial culture (Fig. 2). Histological examination revealed no specific findings besides active inflammatory and necrotic tissue. *Providencia rettgeri* and *Enterococcus faecalis* were detected in a bacterial culture test. T2 image of the magnetic resonance imaging performed at the time of admission revealed some necrosis of the vastus lateralis muscle, including the subcutaneous layer below the ulcerative lesion of the skin (Fig. 3). Finally, the *O tsutsugamushi* accompanying cellulitis was diagnosed based on physical examination, past history, laboratory findings, magnetic resonance imaging and antibody test. Oral antibiotic of 250 mg azithromycin known to be effective against Tsutsugamushi and intravenous antibiotics, 500 mg of Prepenem (500 mg of imipenem/500 mg of cilastatin) for *P rettgeri* and *E faecalis* were used. Wound management was performed through serial debridement and betadine gauze dressing. Subsequently, negative pressure wound therapy was applied on wound 3 times a week. A reconstructive surgery was planned. Because necrosis was wide and involved the vastus lateralis muscle, a large myocutaneous flap was required to cover the defect. A lateral femoral circumflex artery perforator-based myocutaneous keystone flap was planned to cover the defect when the infection had resolved, and the wound stabilized.

**Figure 4.**
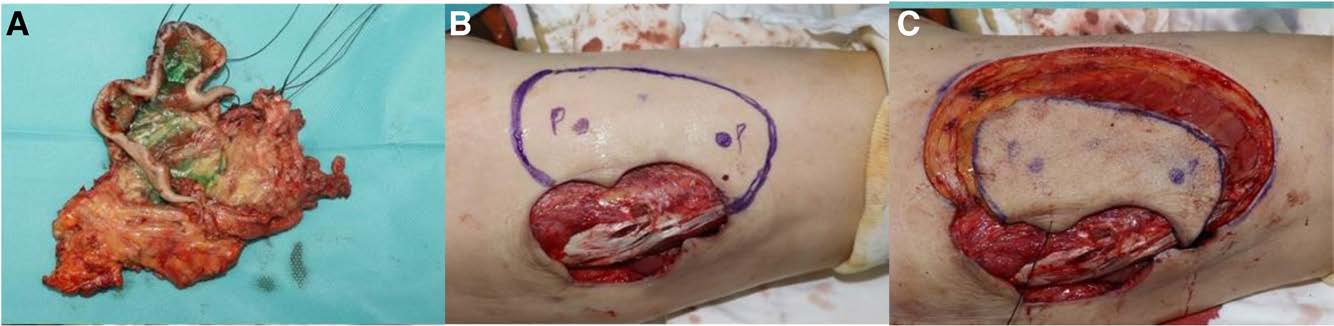
Intraoperative photographic finding. (A) The necrotic tissue was excised by debridement. (B) The myocutaneous keystone flap involving two lateral femoral circumflex artery perforators was designed at the medial side of the defect. (C) The flap was elevated, preserving the two LFCA perforators. LFCA = lateral femoral circumflex artery.

**Figure 5.**
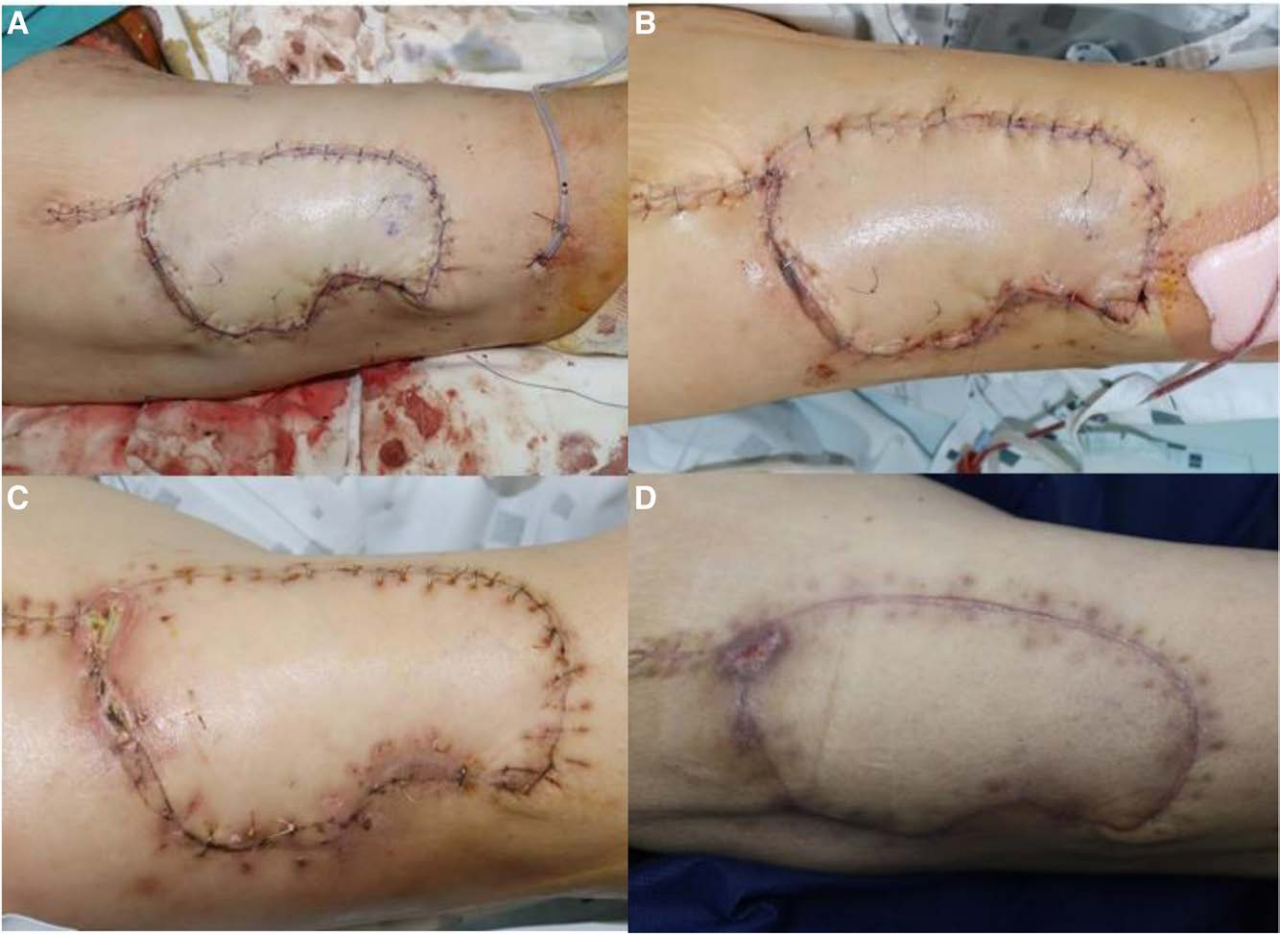
Postoperative photographic finding. (A) Immediate postoperative photographic finding. (B) Postoperative day 2, the flap was in a healthy state except for partially ecchymotic change. (C) Postoperative day 14, the upper margin of the flap was partially necrotic. (D) The wound was totally healed at two months postoperative follow-up.

Under general anesthesia, the remaining necrotic tissue was excised completely, and the lateral femoral circumflex artery perforator was confirmed on the medial side of the wound using a hand Doppler. After identifying the 2 perforators, a 12 × 8 cm keystone flap containing these 2 perforators was designed. Under meticulous dissection, the myocutaneous flap was elevated (Fig. 4). After inserting the elevated flap into the defect site, two 200 cc Hemovacs were placed under the flap. Wound closure was done with 3 to 0 Vicryl, 4 to 0 Nylon, and a skin stapler. On postoperative day 2, the flap was well maintained with intact Doppler sound on 2 perforators except for partial ecchymotic changes of the proximal portion of the flap. On postoperative day 14, the flap was in a healthy state with partial necrotic change, and the patient was discharged. After 2 months, the wound healed entirely without complications (Fig. 5). As a result, the remaining small eschars recovered spontaneously, the large eschar that caused necrosis was successfully treated, and all other clinical symptoms improved. At 3 years of follow-up, there were no recurrences or any complications.

## 4. Discussion

Several studies have shown that climate change and lifestyle changes can cause infectious diseases in many places and countries, not limited to first-identified habitats of infectious pathogens.^[1]^ Among those infectious diseases, Tsutsugamushushi is known to occur not only in existing Asian-pacific areas and northern Australia but also in Africa, southern Chile, and the middle east.^[4]^ In some parts of Southeast Asia, Scrub typhus accounts for 23% of febrile Hospital admissions.^[11]^ Tsutsugamushi disease is mainly transmitted by chigger bites, usually while working in agricultural fields or outdoors.^[8]^ Fever, headache, anorexia, and myalgia are usually mild. Its clinical course is self-limited.^[7]^ Other signs and symptoms of scrub typhus include nausea, vomiting, diarrhea, generalized lymphadenopathy, and relative bradycardia.^[12]^ However, they can sometimes be fatal, leading to multiorgan failure, including myocarditis, acute respiratory syndrome, and acute renal failure.^[9,13,14]^ Unless there is a high level of suspicion, diagnosis can be missed as its clinical features are nonspecific.^[15]^ In this case, the patient had no clinical symptoms such as fever, headache, or myalgia at admission. However, multiple eschars were found at admission to our hospital. In general, its symptoms are known to last 1 to 3 weeks.^[16]^ The patient came 2 weeks after the onset of symptoms. Thus, it was likely that her symptoms had disappeared.

Laboratory findings such as elevated alanine transaminase/aspartate transferase or leukopenia/leukocytosis are nonspecific. Laboratory-based diagnosis of scrub typhus can be made by performing serological tests such as indirect immunofluorescence assay (IFA), enzyme-linked immunosorbent assay, and polymerase chain reaction.^[14,17]^ However, there are many false-negative results in the acute phase. In most cases, diagnosis and treatment are based on clinical symptoms. The patient showed positive results of the *O tsutsugamushi* antibody test, a qualitative test. However, antibody titers, immunoglobulin (Ig) M antibody, 1:16, and IgG antibody, 1:128, using the IFA test were assessed for confirmation. In the case of the IFA test, there is no consensus on the cutoff antibody titer. Nevertheless, based on the diagnostic criteria in South Korea, tsutsugamushi disease can be confirmed when the antibody titer rises more than 4 times between that measured in the acute phase and that in the convalescent serum, or when the IgM antibody titer is 1:16 or higher, or when the IgG antibody is 1:256 or higher in a single serological test.^[8,14,18]^ Tsutsugamushi disease could not be differentiated in this case at admission because the patient came to the hospital with an unusually large eschar for more than 2 weeks after the first eschar occurred. In addition, the IFA test was performed after hospitalization. Hence, it was thought that the test was performed when the acute phase of the disease had passed. Therefore, it could be considered that the antibody titer of IgG decreased, resulting in a false-negative.

At the site of the chigger bite, an eschar with central necrosis can be seen.^[19]^ Eschar is one of the clinical features of Tsutsugamushi. It has been found in 79.5% of patients.^[20]^ It is common in the chest and abdomen in women (42.3%) and the armpit and groin in men (55.8%).^[21]^ In other studies, it occurred most prevalently in women in the front chest above the umbilicus (40.7%) and in the lower abdomen and groin below the umbilicus (24.1%). In men, the lower umbilicus (35.8%) was the most prevalent, followed by the lower extremities (22.6%) and front chest above the umbilicus (20.8%).^[20]^ In this case, an 8 × 6 cm-sized large eschar was on the right thigh, which was not a typical size or location. In most cases of tsutsugamushi disease, the eschar is 5 to 20 mm in size. The eschar naturally falls off and heals. However, in the present case, there was extensive eschar with a debritic change.^[7,19]^ In our case, in addition to a large eschar on the right thigh, multiple small eschars were found on the head and flank, where were not common locations of eschar. In a previous study, eschar was found in the lower limbs (7.4%), the head (4.6%), and the flanks (24.1%).^[20]^

Various complications can occur at the Chigger bite site, rarely with cellulitis.^[22,23]^ In most cases, Tsutsugamushi disease can be treated conservatively. Most of its symptoms can be controlled with an oral antihistamine or topical corticosteroids.^[24]^ Mild symptoms usually improve on their own within a few weeks. Although the probability is low, a secondary infection can lead to inflammation or infectious complications.^[5,22]^ Complicated scrub typhus can be treated using doxycycline, azithromycin, chloramphenicol, tetracycline, macrolide, and rifampin.^[16,23]^ However, without proper treatment, the mortality rate can go as high as 30%.^[25]^ In our case, necrosis had progressed from the small eschar to extensive soft tissue necrosis. *P rettgeri* and *E faecalis* were identified in the bacterial culture test performed after debridement. *P rettgeri* is commonly found in water and soil as a nosocomial pathogen in immunosuppressed patients.^[26]^ *E faecalis* is one of species of enterococci species. It can cause a variety of infections. Other microorganisms are frequently isolated from the same site for some of these infections. In those situations, it is often unclear whether manifestations of infection result from enterococci or whether these relatively avirulent and opportunistic organisms are merely bystanders or are playing a minor role in the infection.^[27]^ Although this patient had a history of diabetes mellitus and hypertension, it was unrelated to immunosuppression. However, uncontrolled diabetes might have exacerbated the infection to some degree. Bacterial infection can also worse necrosis progression to the muscle layer. However, an appropriate antibiotic was selected based on the results of antibiotic susceptibility testing with wound management in parallel, leading to early wound infection control.

Due to a nonspecific clinical course, unusual size, and an unusual location of eschar with deep tissue necrosis, Tsutsugamushi was ignored initially. Therefore, through this case, we discussed why Tsutsugamushi disease was not diagnosed at an early stage and worsened. This case has several limitations. First, the exact cause and mechanism of why the eschar in the thigh became larger than the usual eschar could not be proven. Second, eschars on the head and side were similar in size to a normal eschar. However, they did not heal after 3 weeks. The cause that delays wound healing was not found. Third, *P rettgeri* and *E faecalis* were detected in the wound. It was impossible to determine whether they were infected from the beginning or during treatment at another hospital. Fourth, the titer of the tsutsugamushi test result might be a false positive. Although the possibility of a false positive cannot be ruled out, it is nevertheless a case in which atypical tsutsugamushi disease was suspected based on the patient’s occupation and season, clinical manifestations, and blood test results.

## 5. Conclusion the limitations of the study were described in detail

In conclusion, when a patient with an eschar comes to a hospital, it is essential to conduct a careful physical examination and history taking for diagnosis, especially in patients with uncontrolled diabetes or immunocompromised. Afterward, step-by-step laboratory tests and appropriate treatment should be administered. In addition, the *O tsutsugamushi* infection can occur worldwide. The possibility should always be considered, even if the patient has nonspecific symptoms.

## Acknowledgments

This work was supported by the National Research Foundation of Korea (NRF) grant from the Korean government (MSIT) (2020R1A2C1100891). It was also supported by Soonchunhyang University Research Fund.

## Author contributions

**Conceptualization:** Hwan Jun Choi.

**Data curation:** Hyun Kim.

**Supervision:** Da Woon Lee.

**Writing – original draft:** Je Yeon Byeon.
